# Increased Household Income Improves Nutrient Consumption in Pakistan: A Cross-Sectional Study

**DOI:** 10.3389/fnut.2021.672754

**Published:** 2021-08-10

**Authors:** Nadia Shabnam, Muhammad Azeem Ashraf, Rizwan Ahmed Laar, Rizwana Ashraf

**Affiliations:** ^1^Department of Health Profession Education, National University of Medical Sciences, Rawalpindi, Pakistan; ^2^Research Institute of Education Science, Hunan University, Changsha, China; ^3^College of Physical Education, Hubei Normal University, Huangshi, China; ^4^Department of Education, University of Lahore, Lahore, Pakistan

**Keywords:** nutrients consumption, income elasticity, parametric methods, nutrients-income relationship, Pakistan

## Abstract

The provision of high-quality food is a primary factor in ensuring adequate nourishment and preventing malnourishment-related diseases in Pakistan. This study, therefore, aimed to quantify the impact of income on nutrient consumption in Pakistan, with the hypothesis that income has a primary role in reducing malnourishment in the developing world. To do this, we estimated nutrient–income elasticity—defined as the proportion of change in nutrient consumption in response to a change in income—for total calories, macronutrients, and micronutrients, using the nationally representative Household Integrated Economic Survey data (2010–2011) for Pakistan. Nutrient–income elasticity values were derived using several parametric regression approaches. We also assessed the non-linearity and endogeneity of the relationship. Calorie–income elasticity was found to be significantly different from zero, irrespective of the estimation technique used. Income elasticity for macronutrients and micronutrients was also found to be significantly different from zero, ranging from 0.29 to 0.65. This study, therefore, supports the hypothesis that increased household income likely improves nutrient consumption.

## Introduction

Obtaining sufficient nutrition from food is an essential human need that remains unmet among the majority of people worldwide, especially in developing nations. Both food supply and logistical requirements must be met in order to provide adequate nutrition to whole populations, ensuring that their healthy and active lifestyles are well-supported (1). In order to achieve this, both overall calorie and specific nutrient requirements must be met. Nutrients are biologically necessary for survival, but people may not consider the nutrient composition of food when purchasing food items. Nutrient demand is therefore indirectly included in patterns of food consumption. This is a key concern in public health; therefore, investigating nutrient acquisition and consumption by individuals is vital for informing public health policies, such as providing incentives for the consumption of nutrient-rich food to prevent malnutrition in the overall population.

Achieving a balanced diet in terms of nutritional composition enables individuals to perform required daily activities, and populations to achieve appropriate health standards (2). However, it is widely established in the literature that the demand for food depends on the specific food group, nutritional value, and availability, along with the cultural values, socio-demographic characteristics, preferences, and lifestyles of consumers. Along with these qualitative demands, the quantity of food products that are consumed may also be related to the quantities of nutrients available, and the nutritional quality of the diet of each consumer (3). Thus, in order to achieve a balanced diet, nutritional requirements and diets may vary widely between individuals.

Provision of quality food is one of the primary food security concerns of the Pakistani government. In 2015–2016, the average per capita availability of calories was 2,473 kcal/day, which exceeds the minimum energy requirement for performing daily activities. However, low nutritional intake is still a problem, reflected in the high rates of malnutrition among Pakistani children under the age of 5 years, accounting for 45% of deaths in this group in 2013; in addition, the growth of approximately 9.5 million (44%) children was found to be stunted, and muscle wastage was observed in 3.5 million (38%) of children (4).

Morbidity and mortality are high in Pakistan, which are thought to be a consequence of common malnutrition-related conditions, such as fever and watery diarrhea, measles, and respiratory infections, resulting in impaired immunity (5). It is assessed that almost 800,000 children died every year in Pakistan, wherein 35% of these deaths occur due to malnutrition. The reasons that were suggested for this were lack of balanced diet, improper weaning or early cessation of breastfeeding, and/or low fruit and vegetable intake often considered a likely reason for micronutrient deficiencies (5). This revealed that the chance of death is higher for children suffering from malnutrition compared to children with a balanced diet. People residing in less-developed areas across Pakistan primarily consume millet grain and yogurt (lassi); however, this is insufficient to meet nutrient requirements (6). This could lead to serious developmental issues in children; for example, iodine and iron deficiency have been identified as significant sources of poor cognitive development, primarily in children <2 years of age (7, 8).

Pakistan is primarily an agricultural country, with two-thirds of its population economically dependent on agriculture for work. A large portion of the population lives in rural areas, where the average per capita expenditures are 31% lower than those in urban areas (9). Approximately 40.8 million of 180 million people in Pakistan are malnourished, and alarmingly, approximately one-third of the population does not have access to enough food to achieve adequate nutrition. Malnutrition is, therefore, a reflection of poverty, but it may also impede the economic growth required to escape poverty in developing countries, including Pakistan. This may occur in multiple ways, such as by reducing the life expectancy and therefore expected productive years in newly born children; reducing resistance to disease, thus increasing absence from work; and inhibiting mental and physical development in children, thus decreasing their potential productivity.

An important concept in development economics is that malnutrition can be eradicated only with improvements in income that accompany long-term economic development. This concept is the focus of a significant body of research and is supported by many studies (3, 10, 11), although metabolism and demographic characteristics (such as rural vs. urban populations) may also be important factors in malnutrition (12–14). However, outcomes from research in this area are heterogeneous. Estimated values for calorie–income elasticity, which describes the proportion of change in calorie intake resulting from a change in income, range from near zero (15–19) to almost one (10, 11, 19–26). One study (3) conducted a meta-analysis of 40 empirical nutritional demand studies for a comprehensive overview of this heterogeneity. The link between income and calorie–income elasticity is currently not well-established (3), and the dynamics of calorie consumption in connection to income growth are still debated.

Most studies have so far focused on calories as an indicator of nutrition; however, one study assessed changes in consumption patterns of nutrients in response to price and income changes in the developed and developing world (27). Unlike the majority of reviews presently available, which report on the income elasticity with regard to calories alone, this review estimated income elasticity with regard to calories, macronutrients, and micronutrients, on a comparative basis. Furthermore, determinants of the heterogeneity associated with these estimates were investigated using meta-analysis. This study, therefore, aimed to carry out a similar nutrient demand analysis for Pakistan.

There were two primary objectives for this study. The first was to examine consumption patterns and trends of foods in different food groups in terms of nutrient composition across different income groups in Pakistan. The second was to estimate the extent to which the consumption of calories, macronutrients, and micronutrients changes in response to an increase in household income. A very limited number of previous studies have investigated the income–calorie relationship (28–30) for Pakistan, but none have explored the income–nutrient relationship. To address this, we provided estimates of income elasticity at the household level in Pakistan, not only for total calories but also for key macronutrients (protein, fat, and carbohydrates) and micronutrients (calcium, iron, iodine, zinc, and vitamin A), derived using parametric (linear and non-linear) estimation methods and Household Integrated Economic Survey (HIES) data for Pakistan (31). We also discussed how our results may help policymakers to improve the quality and accessibility of food, thus addressing the significant problems posed by malnutrition in Pakistan.

## Methods

### Data and Nutritional Assessments

This study used data from the HIES, collected between July 2010 and June 2011. This survey includes all areas within four provinces of Pakistan (i.e., Balochistan, Khyber Pakhtunkhwa, Punjab, and Sindh). A two-stage stratified random sampling scheme was adopted in this survey; separate sampling strategies were used for urban and rural areas. Enumeration blocks in urban areas and mouzas/dehs/villages in rural areas were selected at the first stage (defined here as “primary sampling units,” PSUs) from which households were selected at the second stage (defined here as “secondary sampling units,” SSUs). In urban areas, sample PSUs from each stratum were selected using a probability proportional to size (PPS) method, based on households in each enumeration block as a measure of size (MOS). Similarly, in rural areas, the population of each village was taken as the measure of size (MOS) used to select sample villages using a probability PPS method. Overall, 16 households from each rural PSU, and 12 enumeration blocks from each urban PSU, were selected for this survey using a systematic sampling scheme.

The nationally representative sample included in our study thus comprised 16,341 households overall, covering 14 large cities and 81 districts, including both urban and rural areas. The HIES reports information on a variety of social issues, including education, health, employment and income, immunization, use and satisfaction with facilities and services, and details of household consumption. Within the consumption module, the survey collects information on the quantities and values of 69 food items. We used these data (quantities and values of 69 food items) in our analysis.

### Estimation of Food and Nutrient Consumption

Household food intake data were recorded every 14 days (fortnightly), with a 30-day recall period for food items. The fortnightly measure of food consumption was converted to monthly consumption. The quantities and nutritional values for more than 69 food items were recorded and stratified according to their sources, for example, from own production, market purchases, and gifts of staple food received in kind. Expenditure on purchased foods was also recorded.

The present analysis aggregated food items into 11 main groups: milk and milk products, meat, fruit, vegetables, spices, sugar, wheat, rice, pulses, oil, and other foods (formation of food groups is given in Supplementary Table A1). To calculate nutrient consumption from the reported food quantities, we applied conversion factors from the Food Composition Table for Pakistan, provided by Pakistani government (32), which contains data on nutrient contents for various foods items. The quantity of a given nutrient consumed was calculated as follows:

(1) $$\begin{array}{r} N\;=\;\Sigma \theta_{i}Q_{i} \end{array}$$

where *N* is the quantity of the nutrient, θ*i* is the average nutrient content of a unit of food *i*, and *Qi* is the number of units of food *i* that were consumed (food items given in HIES food consumption sheet are in multiple units, g/kg/L). The food composition table for Pakistan (Table 1) provides the number of calories and nutrients (macro and micro) in 100 g of edible portion and each nutrient has also different units of measurement. We converted this 100 g into per kg unit to hold the consistency in the units of food items consumed. After the extraction of nutrients from food items, we computed the total daily household energy acquisition per capita. On the basis of this information, per capita daily calorie consumption and per capita consumption of nutrients (macro and micro) were computed (last two columns of Table 2). The current expenditure rather than income was used as a measure of household welfare; this is because expenditure tends to be a more dependable estimate of the permanent income of a household.

**Table 1:** Food composition in Pakistan (100 g of edible portion).

	**Name of food**											
**Sr. # (A)**	**English**	**Urdu**	**Scientific**	**Calories (kcal)**	**Protein g**	**Fat g**	**Carbohydrates g**	**Calcium mg**	**Iron mg**	**Zinc mg**	**Iodine ppm**	**Vit. A RE**
**Milk and milk products**												
1	Milk (buffalo–fluid whole)			105	4.5	7.8	4.4	173	0.2	0.2	-	53
2	Milk (packed by milk plants)			63.6	3	0.03	4.76	131.6	0.08	-	-	0.07
3	Milk (cow–dried whole)			446	25.2	25.1	37.2	938	0.6	3.3	-	280
4	Yogurt			71	3.5	1.2	5.3	166	0.4	0.6	-	30
5	Butter milk	Lassi		31	0.8	1.2	0.6	30	0.8	0.4	-	8
6	Curd	Dahi		52	2.9	3.1	3.3	130	0.3	-	-	0
7	Butter			721	0.8	80.6	1.1	25	0.2	0.1	26	754
8	Cheese	Paneer		35	22.4	26.7	4.2	545	2	3.1	27	278
9	Cream	Balai		361	2.3	38.9	2.2	60	0.1	-	7	252
·	·	·	·	·	·	·	·	·	·	·	·	·
·	·	·	·	·	·	·	·	·	·	·	·	·
108	Wheat Bread	Puri	*Triticum aestivum*	293	8.6	9.1	44.3	17	2.7	1.6	-	0

*Units of measurement for calories and nutrients (macro and micro) are given as kcal, kilocalories; g, grams; mg, milligrams; ppm, parts per million; and RE, retinol equivalent*.

**Table 2:** Computational details of household nutrient consumption.

**Household Code**	**Province**	***R***	**q1101**	**q1102**	**q1103**	**q1104**	**q1105**	**c1101**	**c1102**	**c1103**	**c1104**	**c1105**	**HHsize**	**Total daily calories**	**PCDCC**	**PCDPC**
10011100101	Punjab	1	0	14	1,500	1.5	0	0	8,960	6,690	795	0	9	16439.51	2012.13	47.30
10011100102	Punjab	1	0	10	0	1	0	0	6,400	0	530	0	7	13302.99	2217.17	50.41
10011100103	Punjab	1	0	7	0	2	250	0	4,480	0	1,060	930	6	9220.70	1623.12	36.54
10011100104	Punjab	1	0	5	0	1	0	0	3,200	0	530	0	8	10585.23	1433.73	30.43
10011100105	Punjab	1	0	6	0	1.5	0	0	3,840	0	795	0	6	10316.69	1939.54	43.06
10011100106	Punjab	1	0	8	0	1	0	0	5,120	0	530	0	5	7642.72	1589.42	36.83
10011100107	Punjab	1	0	10.5	0	0	0	0	6,720	0	0	0	6	12467.12	1921.16	52.11
10011100108	Punjab	1	7	0	0	0	0	7,070	0	0	0	0	2	4146.62	1694.70	60.20
10011100109	Punjab	1	4	0	0	0	0	4,040	0	0	0	0	4	9490.07	2197.21	61.36
10011100110	Punjab	1	14	0	0	5	0	14,140	0	0	2,650	0	10	14089.39	1476.81	35.57
10011100111	Punjab	1	16	0	0	0	0	16,160	0	0	0	0	8	13352.44	1700.71	43.97
10011200101	Punjab	2	0	14	0	0.5	0	0	8,960	0	265	0	5	9959.23	2044.03	49.46
10011200102	Punjab	2	0	0	1,500	0.5	0	0	0	6,690	265	0	6	9864.93	1847.22	39.72
10011200103	Punjab	2	0	20	0	2	200	0	12,800	0	1,060	744	9	16489.03	1906.95	45.00
10011200104	Punjab	2	0	7	0	2	0	0	4,480	0	1,060	0	2	5942.22	2765.19	75.25

*This table shows the mechanism which was used to compute the calories and nutrients from raw data by using food composition table. q1101–q1105 is the quantities of food items and c1101–c1105 is the amount of calories in the corresponding food items for each household. HHsize, household size; PCDCC, per capita daily calorie consumption; and PCDPC, per capita daily protein consumption. We provide the computational details of one food group (milk and milk products given in Table 3) for some households for calories and protein, rest of macronutrients (fat and carbohydrates) and micronutrients (calcium, iodine, iron, zinc, and vitamin A) are computed in a similar way*.

**Table 3:** Item-wise food consumption profile of a household in Household Integrated Economic Survey (HIES) consumption module.

**Household Code**	**Province**	***R***	**Itc (Code) Unit**	**q1**	**v1**	**q2**	**v2**	**q3**	**v3**	**q4**	**v4**	**T_Q**	**T_V**
10011100101	Punjab	1	Milk (packed by milk plan) (1,101) L	14	840	0	0	0	0	0	0	14	840
10011100101	Punjab	1	Milk, powdered (for adults) (1,103) g	1,500	645	0	0	0	0	0	0	1,500	645
10011100101	Punjab	1	Curd/yogurt /lassi (1,104) kg	1.5	84	0	0	0	0	0	0	1.5	84
10011100101	Punjab	1	Other foods like ferni, kheer, custard (1,106)	0	130	0	0	0	0	0	0	0	130
10011100101	Punjab	1	Beef (1,201) kg	2	520	0	0	0	0	0	0	2	520
10011100101	Punjab	1	Eggs (1,204) No.	12	75	0	0	0	0	0	0	12	75
10011100101	Punjab	1	Banana (1,301) No.	24	110	0	0	0	0	0	0	24	110
10011100101	Punjab	1	Potato (1,501) kg	5	200	0	0	0	0	0	0	5	200
10011100101	Punjab	1	Onion (1,502) kg	5	190	0	0	0	0	0	0	5	190
10011100101	Punjab	1	Karela, ladyfinger, brinjal (1,505) kg	3	110	0	0	0	0	0	0	3	110
10011100101	Punjab	1	Other (green chillies, tura) (1,509) kg	1	34	0	0	0	0	0	0	1	34
10011100101	Punjab	1	salt simple (rock and sea) (1,601) kg	1	12	0	0	0	0	0	0	1	12
10011100101	Punjab	1	Chillies, red (1,603) g	250	60	0	0	0	0	0	0	250	60
10011100101	Punjab	1	Turmeric, coriander seeds (1,604) g	250	60	0	0	0	0	0	0	250	60
10011100101	Punjab	1	Ginger (1,605) g	250	40	0	0	0	0	0	0	250	40
10011100101	Punjab	1	Garlic (1,606) g	300	48	0	0	0	0	0	0	300	48
10011100101	Punjab	1	Cinnamon, caraway, cardamom (1,607) g	125	50	0	0	0	0	0	0	125	50
10011100101	Punjab	1	Sugar (desi or milled) (1,701) kg	5	275	0	0	0	0	0	0	5	275
10011100101	Punjab	1	Confectionery (toffee, chocolate) (1,704) No.	50	120	0	0	0	0	0	0	50	120
10011100101	Punjab	1	Squashes and syrups(not medicated) (1,802) L	1	125	0	0	0	0	0	0	1	125
10011100101	Punjab	1	Wheat and wheat flour (2,101) kg	60	1,800	0	0	0	0	0	0	60	1,800
10011100101	Punjab	1	Rice and rice flour (2,102) kg	10	600	0	0	0	0	0	0	10	600

*R, region (region = 1 for urban and 0 = rural), Itc, food items consumed, q1, q2, q3, and q4 indicate the sources from which the household acquired the food. v1, v2, v3, and v4 indicate the values or expenditure on acquired food items. There are two main sources: paid and consumed and unpaid and consumed. q1 and v1 belong to paid and consume category (those goods and services that actually consumed by the household and distinguished from total household purchases). Similarly, q2, q3, and q4 and v2, v3, and v4 belong to unpaid and consume category (those goods and services which are received as wages and salaries in kind, own produced goods and services, and received in the form of gifts, assistance, inheritances, or other sources, respectively). T_Q is the total food quantity consumed and T_V is the total value of respective food quantities. This table shows the consumption and expenditure profile for only one household of Punjab belonging to urban area. We have total 16,341 households in 2010–2011*.

### Estimation of Nutrient Income Elasticities

#### Linear Specifications

The principal hypothesis to be tested in this study was that increased income is related to per capita nutrient consumption. For this purpose, we chose a linear specification of parametric form, which assumed that the conditional relationship between income and nutrient consumption is linear; in other words, the income elasticity of nutrient demand is constant. For each nutrient, the following equation was used:

(2) $$\begin{array}{r} ln{Nut}_{i}\;=\;\beta_{0}+\theta lnX+\beta_{1}Z+\epsilon_{i} \end{array}$$

where *Nut*_*i*_ is the per capita nutrient consumption of household *i, X* is the per capita total expenditure (PCE) of household *i, Z* is a matrix of household characteristics including household size, age, and education level of the head of the household, education level of women, employment status, age–gender household composition ratios, agricultural status of the household, possession of livestock, and water and sanitation facilities; regional and provincial dummies are also included in this regression model, and ε_*i*_ is an error term (details about these variables are given in Supplementary Table A2).

We checked the normality of the dependent variable by using the Kolmogorov-test (*p*-value = 0.000) and the Shapiro–Wilk test (below 0.05). The logarithmic transformation was chosen because the empirical joint density of the logarithms is a good deal closer to joint bivariate normality than for any other obvious data transformation, so that the regression functions are close to being linear. Furthermore, if normality is accepted, at least as an approximation, other functions can be calculated, most obviously the regression function of calories and other nutrients on income. In addition, we used the same test (Kolmogorov and Shapiro–Wilk test) to test the distribution of all the variables. Some of them were positively skewed and some negatively skewed depending mostly on the nature of the variables. The logarithmic transformation was applied only on income and price variables. We checked that residuals were approximately normal.

We measured nutrient consumption by converting food quantities into available nutrients. This method assumes no food is wasted, or given and received outside of the household. This may cause a source of bias. Other factors may also result in biased estimates, and unobserved variables may be correlated both with expenditure and nutrient consumption. The total expenditure as used in the analysis has usually measurement errors and these errors of measurement may be positively correlated with those of measurement errors in nutrients. This type of correlation between the measurement errors in the dependent and independent variables in a regression analysis means that this is not a standard error-in-variables problem (8, 11). However, these sources of bias can originate the endogeneity problem, and estimation of Equation (2) by linear regression does not take into account any potentially imperative sources of bias.

To account for this, we estimated Equation (2) using an instrumental variable (IV) method. The choice of a valid instrument is based on its relevancy and exogeneity. We chose two different instruments: non-food expenditure (8, 11, 22) and square of non-food expenditure (8).

### Non-linear Specifications

Given the possible non-linear relationship between income and calorie intake, we used two different approaches to assess this. First, we used a flexible specification, in which the parameters were linear but which permits elasticity to vary with income. According to Engel's law, as income increases, the proportion of income spent on food items diminishes. Thus, it is expected that as income rises, the calorie–income elasticity also decreases.

(3) $$\begin{array}{r} {lnNut}_{i}=\beta_{0}+\theta_{0}\ln X_{i}+\theta_{1}{\left(\ln X_{i}\right)}^{2}{+\beta }_{1}Z_{i}+\varepsilon_{i} \end{array}$$

Differentiating Equation (2) with respect to ln *X*_*i*_ yields:

(4) $$\begin{array}{r} \frac{\partial lnNut_{i}}{\partial lnX_{i}}\;=\;{\;\theta }_{0}+{2\theta }_{1}\ln\;X_{i} \end{array}$$

(5) $$\begin{array}{r} {lnNut}_{i}\;=\;\beta_{0}+\gamma_{0}\ln X_{i}+\gamma_{1}\frac{1}{X_{i}}{+\beta }_{1}Z_{i}+\in_{i} \end{array}$$

And differentiating Equation (4) with respect to ln *X*_*i*_ yields:

(6) $$\begin{array}{r} \frac{\partial ln{Nut}_{i}}{\partial lnX_{i}}=\gamma_{0}-\frac{\gamma_{1}}{X_{i}} \end{array}$$

We obtained estimates using Equations (3) and (5) for each nutrient. If the coefficients attached to per capita expenditure in Equations (3) and (5), θ_1_ and γ_1_, respectively, are significant, then nutrient income elasticities vary with the level of income. For example, in model (5), if γ_0_ > 0 and γ_1_ < 0, then nutrient income elasticity decreases as income rises. If γ_0_ = γ_1_/*X*_1_, the elasticities will be 0 and if γ_0_ = γ_1_/*X*_1_, the elasticities will be negative. Moreover, when γ_1_ = 0, the elasticities become constant and equal to γ_0_ [for a detailed discussion on the advantages of log linear inverse model see (33)].

Finally, we addressed the issue of non-linearity in nutrient–income relationships using non-parametric and semiparametric regression. Non-linearity in the relationship between nutrients and income indicates that relatively less well-nourished individuals are more likely to make larger nutritional changes with budget shifts than those with better nourishment (34). In other words, people with greater economic security gain little nutritional benefits, while those with less economic security gain more from increased nutrient consumption. It is therefore imperative to understand the full range of nutritional responses instead of aggregating them into a single-point estimate. In order to explore the nutrient consumption patterns and deficiencies, it is of greater importance to carry out summary statistics of sampled households. Other issues such as the behavior of nutrient consumption to income changes are discussed in the section next to summary statistics.

## Results

### Descriptive Analysis

Food consumption patterns of an individual or household indicate the composition of the available food, consumed by the population as a whole. In Pakistan, food consumption patterns generally indicate a greater risk of malnutrition. Addressing the first objective of this study, Tables 4–**7** show the increase or decrease in average consumption for key nutrients (macro and micro) compared with the average recommended intake, and the contribution of each food group in the per capita daily intake of calories and nutrients. We have also observed the contribution of each food group in average calorie consumption at different income levels.

**Table 4:** Percentage share of calories and nutrients in food groups.

**Food groups**	**Calories**	**Protein**	**Fat**	**Carbohy**	**Calcium**	**Iron**	**Iodine**	**Zinc**	**Vitamin A**
	**(kcal)**	**(g)**	**(g)**	**drates (g)**	**(mg)**	**(mg)**	**(μg)**	**(mg)**	**(μg RE)**
Milk	10.69	17.46	25.24	2.82	65.04	4.11	0.36	5.12	24.81
Meat	2.63	9.25	6.81	0.03	1.54	2.32	13.13	4.22	3.26
Fruit	2.04	1.13	0.31	3.1	2.35	1.56	16.1	1.06	9.38
Vegetables	3.19	4.16	0.47	4.34	7.37	5.61	64.68	3.77	26.33
Spices	0.5	0.74	0.31	0.55	2.18	1.79	0	1.06	0.37
Sugar	11.33	1.2	8.56	17.61	1.05	34.94	2.1	0	0.38
Wheat	45.9	49.5	5.05	58.95	15.4	43.67	0	74.83	0
Rice	5.93	4.25	0.48	7.98	1.11	1.87	0	3.99	0
Pulses	2.23	5.69	0.81	2.4	3.27	3.32	0.4	4.37	0.55
Oil	13.82	0.14	51.19	0	0.09	0	0	0	34.5
Other food groups	1.02	5.43	0.59	1.25	0.43	0.51	0.71	0.75	0.04

The consumption patterns in Table 4 indicate the low diversification of the diet of people in Pakistan: cereals were found to constitute a major part of their diet, within which wheat and wheat products alone provide 46% of the total daily per capita calories consumed. On average, 50% of total protein intake was found to come from wheat consumption. Wheat was also a significant source of carbohydrate (contributing 59% of total carbohydrate intake), iron (44% of total iron intake), and zinc (75% of total zinc intake). Pulses contributed 6% of the total protein intake. Overall, the consumption of animal protein in our sample was relatively low, at 6.4 g per person per day, with households obtaining the majority of their protein from cereals. The results of the study are consistent with a previous study (3) in which cereal was found to provide 54% of total protein intake, with low consumption of high-protein foods, such as animal products, when considered as a proportion of total protein. This indicates that the quality of the diet of many Pakistanis is nutrient-poor.

Fruits and vegetables accounted for 9 and 26%, respectively, of total vitamin A consumption; this is low compared with the recommended intake. Low intake of animal products, fruits, and vegetables often results in micronutrient deficiencies (5). Table 5 shows the mean calorie consumption from different food groups in the 25% of households with the lowest and highest incomes, respectively. These data show that households with the highest incomes obtain more calories from expensive food items including milk and meat compared with the lowest income households, who obtain most of their calories from wheat.

**Table 5:** Average calories consumption from each food group at different income levels.

**Food groups**	**Average calorie consumption of** **(bottom 25 %)**	**95% CI** **(LL, UL)**	**Average calorie consumption of** **(Top 25 %)**	**95% CI** **(LL, UL)**
Milk	116.0357	113.423, 118.65	379.0401	370.62, 387.47
Meat	21.8167	21.203, 22.431	107.5021	105.103, 109.901
Fruit	17.2203	16.281, 18.159	85.8333	82.782, 88.884
Vegetables	48.90382	48.202, 49.606	84.3958	83.123, 85.674
Spices	6.3402	6.195, 6.485	15.372	15.062, 15.652
Sugar	177.4603	174.404, 180.516	293.8418	288.286, 299.398
Wheat	844.4921	835.0845, 853.90	759.542	750.053, 768.031
Rice	94.9373	90.397, 99.477	142.3725	138.133, 146.613
Pulses	33.7731	31.487, 36.358	63.131	61.896, 64.366
Oil	205.1743	203.005, 207.343	386.9707	381.999, 391.9421
Other food groups	6.9315	5.978, 7.884	55.3241	50.664, 59.984

Along with calculating patterns of nutrient consumption overall, to explore the association between income and nutrient consumption patterns, we also created two further groups in our analysis based on income: the 25% of households with the lowest expenditure and the 25% of households with the highest expenditure. Although for very poor households differences might be less noticeable in current expenditure and current income (8). Table 6 shows the mean, median, and interquartile range for calories, major macronutrients (protein, fat, and carbohydrates), and micronutrients (calcium, iron, iodine, zinc, and vitamin A). In order to calculate calories, we included only households with a per capita calorie consumption of 600–8,000 kcal inclusive. The value of the mean was found to be greater than the median for total calories and all nutrients (macro and micro). This indicates that the distribution of all nutrients was positively skewed, and consideration of the mean alone would have resulted in an overestimation of nutrient consumption; the median, interquartile range (IQ = Q7525–Q25), and significance in nutrient intake for low- and high-income groups are therefore also reported (Table 6).

**Table 6:** Mean, median, and interquartile range (IQR) of per capita daily consumption of nutrients (PCE) for all households, the lowest 25% of households by income, and the highest 25% of households by income.

**Nutrients**	**Overall**			**Lowest 25% of households**			**Highest 25% of households**			**Mean interquartile difference**
	**Mean**	**Median**	**IQR**	**Mean**	**Median**	**IQR**	**Mean**	**Median**	**IQR**	
Calories	2,227	2,125	777.45	1,752.56	1,731.59	461.8	2,738	2,633	956.26	−70.45 (14.021)^***^
Protein	55.34	52.26	21.83	40.02	39.46	11.78	74.07	69.99	26.56	−87.87 (0.391)^***^
Fat	65.71	59.11	32.87	42.59	41.29	15.39	95.49	88.23	40.32	−84.05 (0.633)^***^
Carbohydrates	348.38	333.03	129.26	278.26	274.4	86.33	417.23	398.54	166.86	−59.14 (2.382)^***^
Calcium	578.93	482.91	358.99	341.21	324.55	167.44	899	784.05	509.43	−70.08 (8.007)^***^
Iron	28.28	21.16	13.71	21.91	17.49	11.08	35.81	25.13	17.07	−22.38 (0.623)^***^
Iodine	54.36	44.09	35.66	32.98	28.66	18.65	80.91	67.14	49.34	−55.56 (0.858)^***^
Zinc	10.63	10.14	4.07	8.42	8.25	2.97	12.76	12.15	4.94	−58.16 (0.076)^***^
Vitamin A	457.17	392.08	283.51	293.54	272.09	137.82	652.65	577.55	392.35	−62.09 (5.866)^***^

*Samples included only households with calorie consumption of between 600 and 8,000 kcal/day. Values in bold show nutrient deficiency in relation to recommended values. Standard errors are given in parentheses; t-tests were used to determine statistical differences, with*

*** *p < 0.01*.

The mean per capita daily calorie consumption was 2,227 kcal in the study period (2010–2011), and the households with the highest incomes had a mean calorie consumption approximately 36% higher than those with the lowest incomes. The greatest difference in the consumption of any specific nutrient between these income groups was for calcium, with high-income households consuming 62% more than lower-income households. Calcium is a vital micronutrient, and deficiency may result in bone or tooth weakness and serious conditions such as osteoporosis. The high prevalence of osteoporosis in Pakistan owing to inadequate calcium intake in the older population is a significant public health issue. Low-income households may therefore be at greater risk of this condition owing to reduced calcium intake observed in our study. Iron consumption was also 39% higher and vitamin A consumption 55% higher in households with the highest vs. lowest incomes. However, on average, iron intake was found to be adequate for the majority of the population according to nutritional guidelines, whereas the consumption of vitamin A was found to be lower than recommended daily allowance. Vitamin A deficiency results in corneal deterioration and childhood blindness in many parts of the developing world, including Pakistan; the results of the study, therefore, highlight this as an important consideration in addressing malnutrition in Pakistan. Other differences in the consumption of specific nutrients were found to be relatively small between the highest and lowest income groups. In our sample, the median person suffers from micronutrient deficiencies (based on a comparison with the recommended daily allowance) in calcium, iodine, zinc, and vitamin A, as well as in overall calories, protein, and fat. Deficiencies were found to be worse in the lowest-income group, which included all macronutrients and micronutrients as well as overall calories. Conversely, the highest-income group was found to be deficient in calcium, iodine, zinc, and vitamin A. These results support our hypothesis that income affects nutrient consumption, with lower-income households experiencing a greater number of deficiencies. The per capita consumption of nutrients in the lower- and higher-income households was found to be significantly different at 1% level of significance (*p* < 0.01). Having information about the prevailing nutrient consumption patterns among these segments of the sample, we further study the nutrient consumption adequacy (Table 7) for the same groups of income distribution.

**Table 7:** Adequacy ratio of nutrient consumption for all households, the lowest 25% of households by income and the highest 25% of households by income.

**Nutrients**	**Reference values**	**All**	**Lowest 25% of households**	**Highest 25% of households**
Calories	2,350 kcal	90.43	73.68*	112.05
Protein	50 g	104.52	78.92*	139.98
Fat	65 g	90.94	63.52*	135.74
Carbohydrates	300 g	111.01	91.47	132.85
Calcium	1,000 mg	48.29*	32.46*	78.41*
Iron	20 mg	105.8	87.45*	125.65
Iodine	15 μg	29.39*	19.1*	44.76*
Zinc	15 μg	67.6*	55*	81.00*
Vitamin A	750 μg (RE)	52.28*	36.28*	77.01*

Adequacy ratio was calculated as the ratio of median intake (as in Table 6) to recommended daily intake. Values with * show nutrient deficiency compared with recommended values.

*RE, retinol equivalents*.

In addition, we calculated nutrient consumption adequacy as the ratio between the median consumption of nutrients and the recommended daily allowance obtained from nutritional tables. An important implication of these results is that individual households in the highest-income group could have a nutrient consumption adequacy >100%, while those of the lowest-income group could be substantially <100%, so remedial interventions for nutrition may be very different for both groups. The data in Table 7 show that the highest-income households had full nutrient consumption adequacy for macronutrients (all >100%), whereas the corresponding values were all below 100% in the lowest-income households, apart from carbohydrates. Some nutrients, such as protein, carbohydrates, and iron, had a strikingly high adequacy ratio in the highest-income group. On average, calorie consumption was 90% of the reference consumption level, but this value was lower for the lowest-income households (74%). Protein consumption did not appear to be a major nutritional issue in our study sample, probably because wheat is a major component of the food consumed in this population, and wheat is a reasonably good source of protein (consistent with the first EDA result in Table 4). Fat consumption was found to be greater than the reference level (135%) for higher-income households, but lower-income households consumed lower than the reference level.

Overall, these data show that total calorie intake may not be a serious threat for lower-income households; however, major deficiencies in key micronutrients, particularly calcium, iodine, zinc, and vitamin A, affect lower-income households to a substantially greater extent than those with higher incomes.

### Models

The ordinary least squares (OLS) estimation results of Equation (2) reported in Table 8 show that estimated income elasticities for all households were significant and positive for calories, all macronutrients, and all micronutrients. These results are in accordance with those from previous studies. For example, calorie income elasticity was found to be 0.38, which is similar to calorie income elasticity estimates for India (0.35) (10), Indonesia (0.43) (35), and rural Mexico (0.44) (8). The nutrient income elasticity for fat, calcium, iodine, and vitamin A were all in the range of 0.54–0.65; these results are consistent with adequacy ratio results, that is, larger elasticity estimates are associated with a lower adequacy ratio (below 100%) of nutrients.

**Table 8:** Nutrient income elasticity estimates: linear specification.

**Nutrients**	**OLS**	**IV Non-food expenditure**	**IV Square of non-food expenditure**
Calories	0.378^**^ (0.006)	0.277^**^ (0.007)	0.269^**^ (0.007)
Protein	0.442^**^ (0.007)	0.337^**^ (0.007)	0.329^**^ (0.007)
Fat	0.575^**^ (0.000)	0.451^**^ (0.009)	0.442^**^ (0.009)
Carbohydrates	0.301^**^ (0.006)	0.217^**^ (0.007)	0.210^**^ (0.008)
Calcium	0.649^**^ (0.011)	0.469^**^ (0.011)	0.461^**^ (0.011)
Iron	0.406^**^ (0.011)	0.341^**^ (0.013)	0.333^**^ (0.013)
Iodine	0.619^**^ (0.011)	0.520^**^ (0.014)	0.517^**^ (0.013)
Zinc	0.285^**^ (0.007)	0.222^**^ (0.007)	0.213^**^ (0.008)
Vitamin A	0.538^**^ (0.011)	0.402^**^ (0.011)	0.393^**^ (0.012)
F test instruments		32758.12	42240.78
Statistical significance (*p*)		0.000	0.000
Observations (*n*)	16290	16290	16290

*Standard errors are given in parentheses; t-tests were used to determine statistical differences, with*

** *p < 0.05. Instruments for ln PCME are ln non-food expenditure and square of non-food expenditure. The Hausman-type test for the absence of endogeneity in the expenditure variable was rejected in all cases. Each column represents a separate regression analysis. The complete set of control variables included in each model is given in the Appendix*.

We estimated Equation (2) with an instrumental variable (IV) two-stage least square (2SLS) method. The choice of a valid instrument, includes the relevancy and exogeneity of the instrument, is always a critical job. We used two different instruments; non-food expenditure and square of non-food expenditure. The results of IV approach are given in Table 8. The value of the *F*-test was >10, indicating that these instruments are valid. Elasticities estimated by IV regression for calories and all macro- and micronutrients were found to be lower in magnitude than the OLS estimates. This pattern indicates upward bias in estimates obtained using the OLS approach. An interesting result is that IV approach also shows a high magnitude (but less than OLS) for those macronutrients and micronutrients for which the magnitude is high in the OLS approach. These results are also consistent with our descriptive result and reveal that improvement in income appears to be most valuable particularly for those key nutrients for which the median person is most deficient.

Ordinary least squares results using specifications 3 and 5, presented in Tables 9, 10, were similar, but the IV results had a different magnitude for these specifications between specifications 3 and 5. Income elasticity was found to be high, sizeable, and significant for fat, calcium, iodine, and vitamin A using both OLS and IV results for specification 3. However, income elasticity for calories was found to be insignificant according to our IV results for specification 5. Intake of carbohydrates, calcium, iron, and zinc did not appear to change with income (IV estimates did not reach significance). In almost all models, per capita expenditure had a significantly positive coefficient.

**Table 9:** Nutrient–income elasticity estimates: non-linear specification with square term (Equation 3).

**Nutrients**	**OLS**	**IV**
Calories	0.460^***^ (0.084)	0.372^***^ (0.204)
Protein	0.523^***^ (0.102)	0.432^***^ (0.204)
Fat	0.687^***^ (0.127)	0.572^***^ (0.219)
Carbohydrates	0.357^***^ (0.115)	0.293^***^ (0.255)
Calcium	0.789^***^ (0.135)	0.575^***^ (0.237)
Iron	0.472^***^ (0.170)	0.435^***^ (0.389)
Iodine	0.708^***^ (0.172)	0.669^***^ (0.389)
Zinc	0.364^***^ (0.126)	0.317^***^ (0.314)
Vitamin A	0.645^***^ (0.165)	0.511^***^ (0.278)
F statistic (first stage regression)		18922.14
*F-*test (log per capita expenditure) *p-*value	2244.70 0.000	
*F-*test (log per capita expenditure)^2^*p-*value	1853.74 0.000	
Observations (*n*)	16290	

*Standard errors are given in parentheses; t-tests were used to determine statistical differences, with*

*** *p < 0.01. Instruments for ln PCME are ln non-food expenditure and square of non-food expenditure. The Hausman-type test for the absence of endogeneity in the expenditure variable was rejected in all cases. Each column represents a separate regression analysis. The complete set of control variables included in each model is given in the Appendix*.

**Table 10:** Nutrient–income elasticity estimates: non-linear specification with inverse term (Equation 5).

**Nutrients**	**OLS**	**IV**
Calories	0.370^***^ (0.012)	0.265 (0.027)
Protein	0.433^***^ (0.015)	0.324^***^ (0.026)
Fat	0.562^***^ (0.021)	0.435^*^ (0.029)
Carbohydrates	0.292^***^ (0.014)	0.205 (0.033)
Calcium	0.634^***^ (0.022)	0.454 (0.034)
Iron	0.398^***^ (0.026)	0.328 (0.053)
Iodine	0.607^***^ (0.025)	0.508^***^ (0.061)
Zinc	0.277^***^ (0.018)	0.207 (0.039)
Vitamin A	0.524^***^ (0.023)	0.386^***^ (0.038)
*F* statistic (first stage regression)	6,792.80	
*F-*test (log per capita expenditure) *p-*value	2,244.70 0.000	
*F-*test (1/per capita expenditure) *p-*value	822.20 0.000	
Observations (*n*)	16,290	

*Standard errors are given in parentheses; t-tests were used to determine statistical differences, with*

* *p < 0.1 and*

*** *p < 0.01. Instruments for ln PCME are ln non-food expenditure and square of non-food expenditure. The Hausman-type test for the absence of endogeneity in the expenditure variable was rejected in all cases. Each column represents a separate regression analysis. The complete set of control variables included in each model is given in the Appendix*.

## Discussion

A variety of estimation procedures was used in the study, by controlling a variety of variables, to demonstrate that nutrient–income elasticity is significant. Our results support the hypothesis postulated in previous studies that (3, 10, 11, 23, 34) any policy aiming to increase household income will also concurrently reduce malnutrition: increases in household income are likely to improve intake of calories and vital nutrients owing to an increase in the consumption of foods with higher nutrient content, such as meat, fruits and vegetables, and dairy products. We found that calorie–income elasticity was statistically significant when estimated using OLS (0.378) and IV with non-food expenditure (0.277) and square of non-food expenditure (0.269, Table 3). These results show that the IV approach notably reduced the coefficient of total expenditure, although it was still positive and significant.

For a flexible parametric non-linear specification, IV results are not significantly dissimilar from OLS. Moreover, our results are consistent with elasticity estimates from previous studies (22). A comparison of OLS and IV estimates for nutrient demand indicates that OLS estimates are likely to be misleading, owing to bias from correlated errors in consumption and nutrient content. In particular, the findings that IV estimates are generally lower than OLS estimates suggest that upward bias may be more important than attenuation bias in this case.

In conclusion, our study demonstrates that increases in household income in Pakistan appear to translate to greater consumption of vital nutrients, likely resulting from changes in the composition of diets toward increased consumption of meats, vegetables, and fruits rather than mostly comprising cereals. Our results also demonstrate that increased income is associated with greater consumption of nutrients in the most susceptible groups. Our nutrient–income elasticity estimates reveal that increases in per capita income are linked with significant and sizeable increases in overall calorie and macronutrient consumption, as well as that of vital micronutrients, specifically calcium, iodine, zinc, and vitamin A.

### Strengths and Limitations

We used nutritional survey data to estimate the nutrient–income elasticity. This enabled us to obtain detailed nutritional data from a very large sample size from diverse regions across Pakistan, which would not have been feasible otherwise. However, using household economic surveys for nutritional assessments has some limitations affecting accuracy. First, respondents may not remember the exact quantities consumed, especially when the recall period is long, as with the 14- and 30-day recall periods used in the HIES. Second, food consumption recall relates to the total food purchased by the household, not all of which is actually consumed by household members. Some may be fed to pets, wasted, or given to visitors or hired helpers. This could lead to an overestimation of actual food intake. Conversely, the receipt of food gifts and absence of some household members during the survey period may lead to underestimation of food intake. Third, we did not measure consumption for individual household members, as the household consumption survey does not report information about the intra-household food distribution; therefore an equal distribution of food among household members must be assumed. Fourth, the data used in this study were from household surveys conducted in 2010 and 2011, thus further studies are required on the household surveys conducted after this. In addition, this study focused on households whose caloric consumption fell between 600 and 8,000 kcal/day. However, owing to the large sample size and comprehensive measurement of specific nutrients as well as overall calorie intake, this study was able to produce realistic estimates and important insights into the overall nutritional status of the Pakistani population.

### Policy Implications

The results obtained in this study point toward some important policy actions that could be taken to improve nutrition in Pakistan. Of primary concern is increasing and upholding income to reduce food insecurity and malnutrition, which we have demonstrated to be associated with improved nutrition, which could be achieved by strengthening the three primary economic areas in Pakistan: agriculture, industry, and services, on a sustainable basis. Another concern is reducing nutrient shortfalls between average consumption and the recommended daily allowance in low-income households. This could be achieved by strengthening subsidization of food, for example, through networks of utility stores to provide discounted food, direct nutrient supplementation programs, or in-kind transfer of food items, price interventions, cash transfer programs, and social safety net programs. Finally, increased income may not reduce malnutrition alone; attention should also be given to the other socioeconomic and environmental factors including safe drinking water, better healthcare, and education. These factors may help improve food utilization.

## Data Availability Statement

Publicly available datasets were analyzed in this study. This data can be found here: https://www.pbs.gov.pk/node/355.

## Author Contributions

NS and MA: conceptualization, supervision, and project administration. NS, MA, and RL: methodology. NS: software, formal analysis, visualization, and writing of the original draft preparation. NS, RL, and RA: validation. MA, RL, and RA: writing, reviewing, and editing. MA: funding acquisition. All authors have read and agreed to the published version of the manuscript.

## Conflict of Interest

The authors declare that the research was conducted in the absence of any commercial or financial relationships that could be construed as a potential conflict of interest.

## Publisher's Note

All claims expressed in this article are solely those of the authors and do not necessarily represent those of their affiliated organizations, or those of the publisher, the editors and the reviewers. Any product that may be evaluated in this article, or claim that may be made by its manufacturer, is not guaranteed or endorsed by the publisher.
