# Supplementary material for: Increased Household Income Improves Nutrient Consumption in Pakistan: A Cross-Sectional Study
Source: Front Nutr. 2021 Aug 10;8:672754. doi: 10.3389/fnut.2021.672754 (PMC8382849; doi:10.3389/fnut.2021.672754)
Supplement: Supplementary file 1 [file Data_Sheet_1.docx]

# Appendix

Table A1: Detailed information of food items in survey round (2010-11) that aggregated to form the 11 food groups

| **S.No** | **Food Groups** | **Food Items** | **Unit** |
| --- | --- | --- | --- |
| **FORTNIGHTLY FOOD ITEMS** | | | |
| **1** | **Milk** | **Milk and Milk Products** |  |
|  |  | Milk (fresh & boiled) | Ltr |
|  |  | Milk (packed by milk plants) | Ltr |
|  |  | Milk, Powdered (for adults & children ) | Gm |
|  |  | Curd / Yoghurt, Lassi ( buttermilk) | Kg |
|  |  | Butter, Margarine, Cream, Cheese | Gm |
| **2** | **Meat** | **Meat Poultry and Fish** |  |
|  |  | Beef | Kg |
|  |  | Mutton | Kg |
|  |  | Chicken Meat | Kg |
|  |  | Eggs | No. |
|  |  | Fish (fresh, frozen, dried)/ Prawns, Shrimps or Crabs ( fresh, frozen, canned ) | Kg |
| **3** | **Fruit** | **Fresh Fruits** |  |
|  |  | Banana | No |
|  |  | Citrus Fruits (Mosummi, Malta, Kinno etc.) | Kg |
|  |  | Apple | Kg |
|  |  | Dates | Kg |
|  |  | Grapes | Kg |
|  |  | Mango | Kg |
|  |  | Other fresh fruits (Pomegranates, Apricot, Jamons, Lemon, Peer, Peach, Plum, Papaya etc.) | Kg |
|  |  | Canned fruits | Gm |
| **4** | **Vegetables** | **Fresh Vegetables** |  |
|  |  | Potato | Kg |
|  |  | Onion | Kg |
|  |  | Tomato | Kg |
|  |  | Cabbage, Cauliflower | Kg |
|  |  | Karaila, Lady finger, Brinjal, Cucumber | Kg |
|  |  | Tinda, Pumpkin, Bottle Gouard | Kg |
|  |  | Radish, Turnip, Carrot | Kg |
|  |  | Peas, Moongra | Kg |
|  |  | Green Chillies, Tural, Lettuce, Kulfa etc | Kg |
|  |  | Canned Vegetables | Gm |
| **5** | **Spices** | **Condiments & Spices (Whole &Powder)** |  |
|  |  | Salt Simple including rock and sea | Kg |
|  |  | Iodised Salt | Kg |
|  |  | Red peppers | Gm |
|  |  | Turmeric, Coriander seed | Gm |
|  |  | Ginger | Gm |
|  |  | Garlic | Gm |
|  |  | Cinnamon, Caraway, Cardamom, Salan masalah, Licorice root, Cumin seeds, Black pepper,Cloves, Mixed condiments | Gm |
| **6** | **Sugar** | **Sugar, Honey and Sugar Preparations** |  |
|  |  | Sugar of both kind that is milled and desi | Kg |
|  |  | Shakar or Gur | Kg |
|  |  | Honey | Gm |
|  |  | Confectionary (Toffee, chocolate and chewing gum etc) | No. |
|  |  | Barfi, Jaleebi, Halwa and other sweetmeats | Kg |
|  |  | Glucose and Energile | Gm |
|  |  |  |  |
| **MONTHLY FOOD ITEMS** | | | |
|  | **Cereals** | **Wheat and Rice only** |  |
| **7** | **Wheat** | Wheat & wheat flour | Kg |
| **8** | **Rice** | Rice & Rice flour | Kg |
| **9** | **Pulses** | **All kind of Pulses including Whole and split as well as Washed and unwashed** |  |
|  |  | Gram Whole (Black and white) | Kg |
|  |  | Dal Channa | Kg |
|  |  | Dal Massh | Kg |
|  |  | Dal Mong | Kg |
|  |  | Dal Masoor | Kg |
|  |  | Other includes Arthar, Sunflower, Soyabean, Garden peas, Chick and Pigeon | Kg |
| **10** | **Oil** | **Edible oil and Fats** |  |
|  |  | Desi ghee | Kg |
|  |  | Vegetable ghee | Kg |
|  |  | Cooking oils and other oils and fats | Ltr |
| **11** | **Other Foods** | **Other Food** |  |
|  |  | Dry fruits and Nuts |  |
|  |  | Raisin, Dates, Apricot (dried ), Other (Almond, Walnut, Chilgoza, Pistachio, Peanuts, Aniseed) | Gm |
|  |  | Non Alcholic Beverages |  |
|  |  | Carbonated beverages | Ltr |
|  |  | Squashes and Syrups | Ltr |
|  |  | Sugarcane Jucies and fruit jucies (fresh and packed), Minneral water etc. | Ltr |
|  |  | Readymade Food, Drinks etc. |  |
|  |  | Readymade junk foods such as snacks, ice creame and instant foods etc. | No. |
|  |  | Tea and Coffee |  |
|  |  | Tea of different kind such as black and green | Gm |
|  |  | Coffee and other supplements like ovaltine, harlics, milo and complan etc. | Gm |
|  |  | Baked and Fried Products |  |
|  |  | Biscuits | Gm |
|  |  | Bread and Ban | No. |
|  |  | Other baked and fried products such as Samosa, Pakora, pastries, Cake and popcorns etc. | No. |
|  |  | Miscellaneous Food Items |  |
|  |  | Jams, Marmalades/ Tomato Ketchup/pulp/ Pudding, Jelly, Pickles, Chatni, Vinegar, Yeast, Ice etc. |  |

**Table A2. Descriptive statistics for survey round 2010-11**

|  | **2010-11** | | |
| --- | --- | --- | --- |
| Variables | **Mean** | | **Standard Deviation** |
|  |  | |  |
| ***Household Income*** |  | |  |
| Per capita total monthly expenditure | 3380.579 | | 2988.284 |
| ***Proportion of Household Members*** |  |  | |
| Household size | 6.410 | 2.591 | |
| Ratio of male members of age 0-4 years to total household size | 0.055 | 0.099 | |
| Ratio of male members of age 5-9 years to total household size | 0.070 | 0.106 | |
| Ratio of male members of age 10-14 years to total household size | 0.064 | 0.100 | |
| Ratio of male members of age 15-55 years to total household size | 0.276 | 0.168 | |
| Ratio of male members of age 56-plus years to total household size | 0.045 | 0.095 | |
| Ratio of female members of age 0-4 years to total household size | 0.057 | 0.100 | |
| Ratio of female members of age 5-9 years to total household size | 0.062 | 0.099 | |
| Ratio of female members of age 10-14 years to total household size | 0.055 | 0.093 | |
| Ratio of female members of age 15-55 years to total household size | 0.277 | 0.151 | |
| Ratio of female members of age 56-plus years to total household size (taken as base category) | 0.040 | 0.100 | |
| ***Household Head Characteristics*** |  |  | |
| Female household head | 0.084 | 0.278 | |
| Age of the household head | 45.999 | 13.122 | |
| Household head has no education | 0.297 | 0.457 | |
| Household head has primary education | 0.635 | 0.481 | |
| Household head has higher secondary education | 0.645 | 0.479 | |
| Household head has graduate and above education | 0.171 | 0.377 | |
| Household head is self employed | 0.190 | 0.392 | |
| Women Education | 0.670 | 0.470 | |
| Agriculture Landowner | 0.080 | 0.271 | |
| Non-agricultural Landowner | 0.021 | 0.142 | |
| Livestock | 0.059 | 0.235 | |
| Access Water | 0.845 | 0.362 | |
| Toilet Facility | 0.139 | 0.346 | |
| ***Average Unit Prices of Food Items*** |  |  | |
| Milk | 41.158 | 7.538 | |
| Meat | 106.988 | 44.640 | |
| Fruit | 14.233 | 5.014 | |
| Vegetables | 31.419 | 2.626 | |
| Spices | 0.357 | 0.403 | |
| Sugar | 56.807 | 11.852 | |
| Wheat | 23.640 | 5.753 | |
| Rice | 47.829 | 13.428 | |
| Pulses | 102.728 | 11.187 | |
| Cooking Oil | 155.969 | 12.582 | |
| Other foods | 2.218 | 2.806 | |
| ***Region*** |  |  | |
| Urban | 0.423 | 0.494 | |
| Rural | 0.577 | 0.494 | |
| ***Provinces*** |  |  | |
| Punjab | 0.431 | 0.495 | |
| Sindh | 0.234 | 0.423 | |
| Khyber Pakhtunkhwa | 0.186 | 0.389 | |
| Baluchistan | 0.149 | 0.357 | |
| **N** | 16290 | | |
